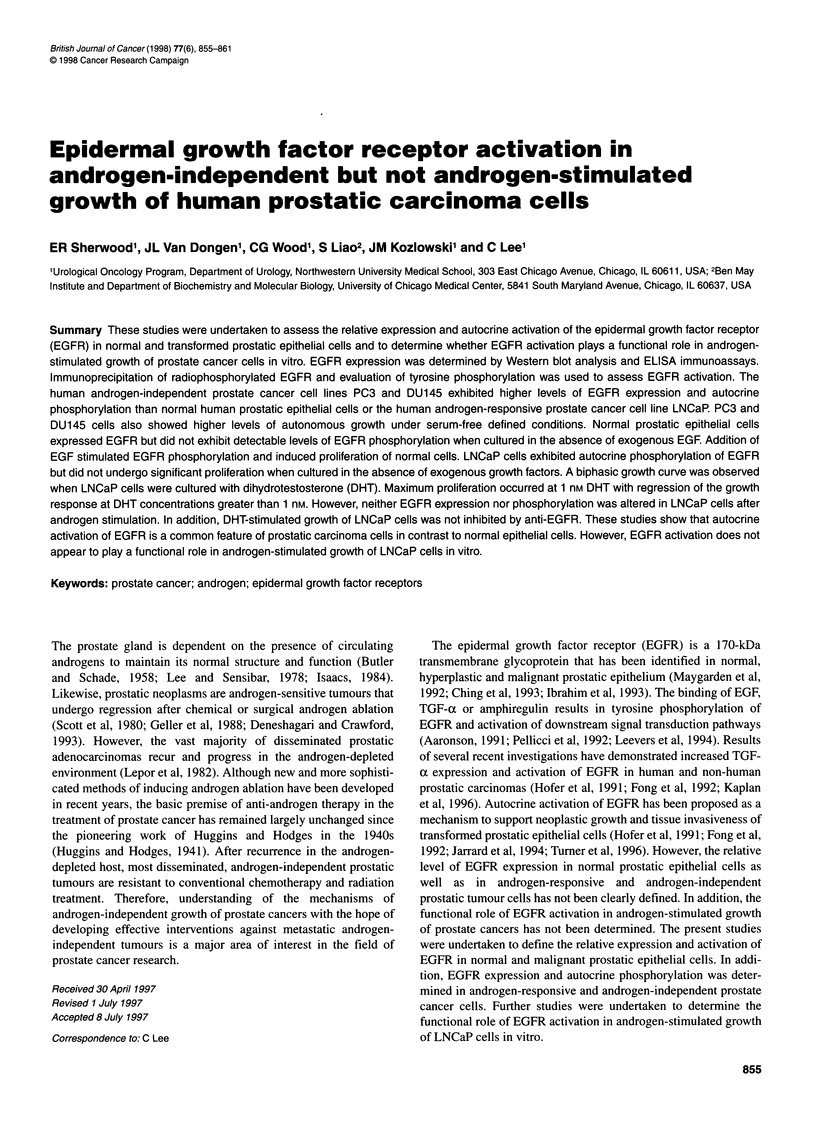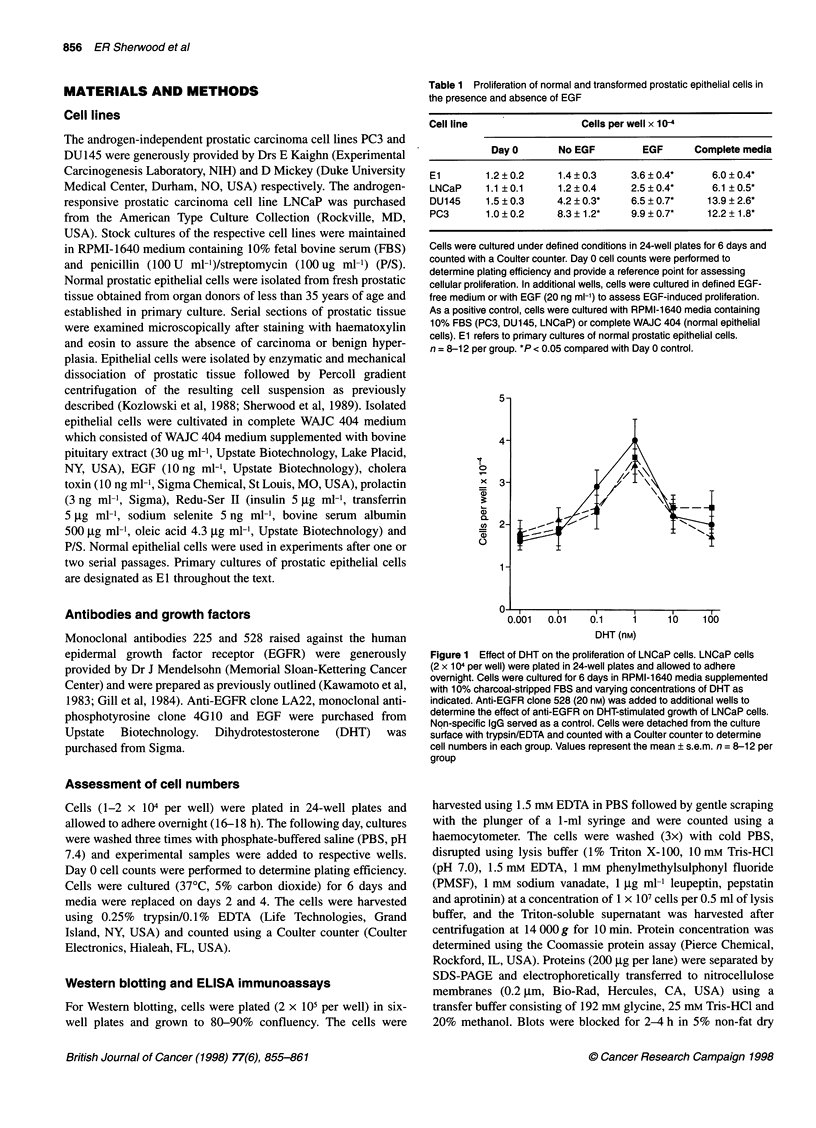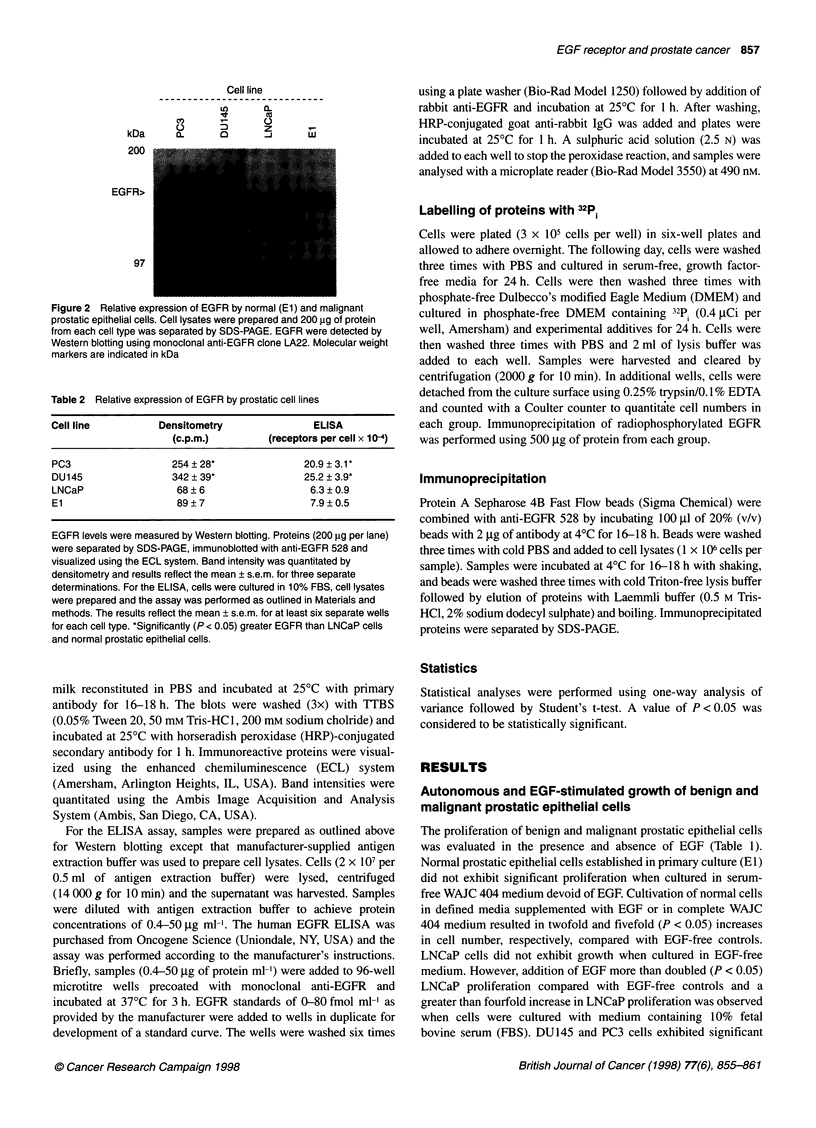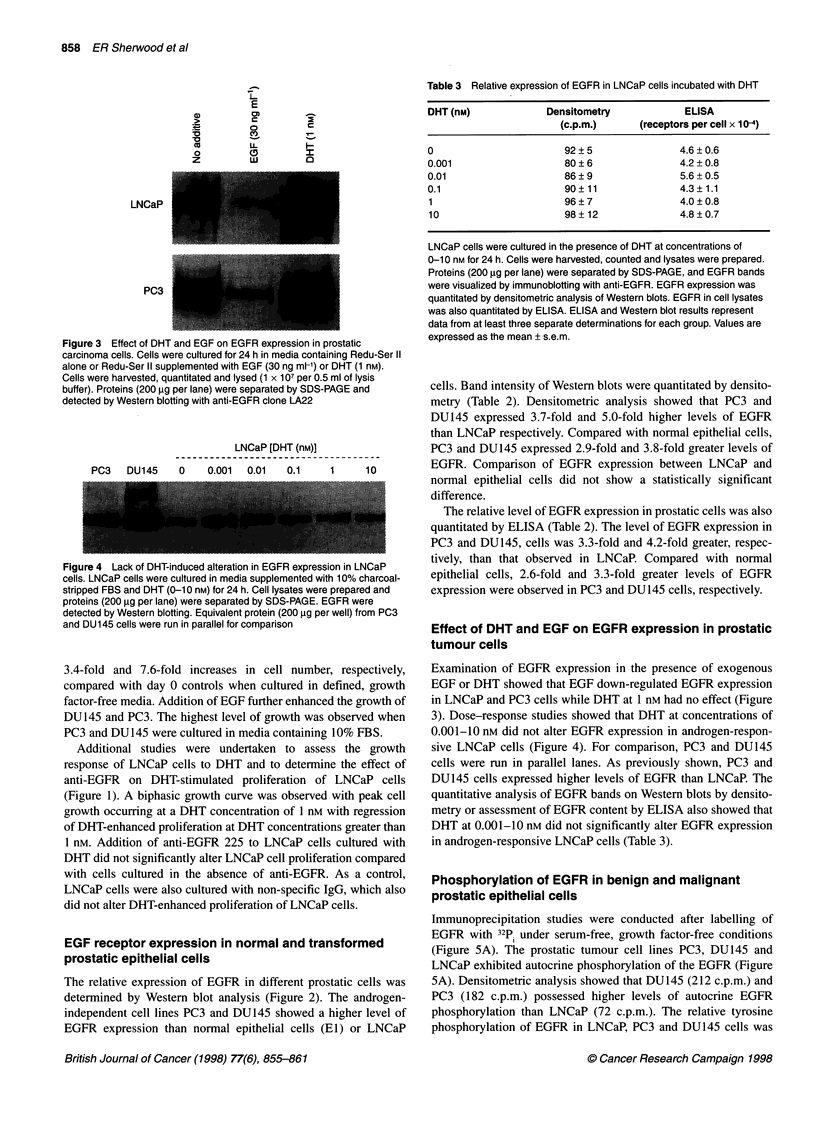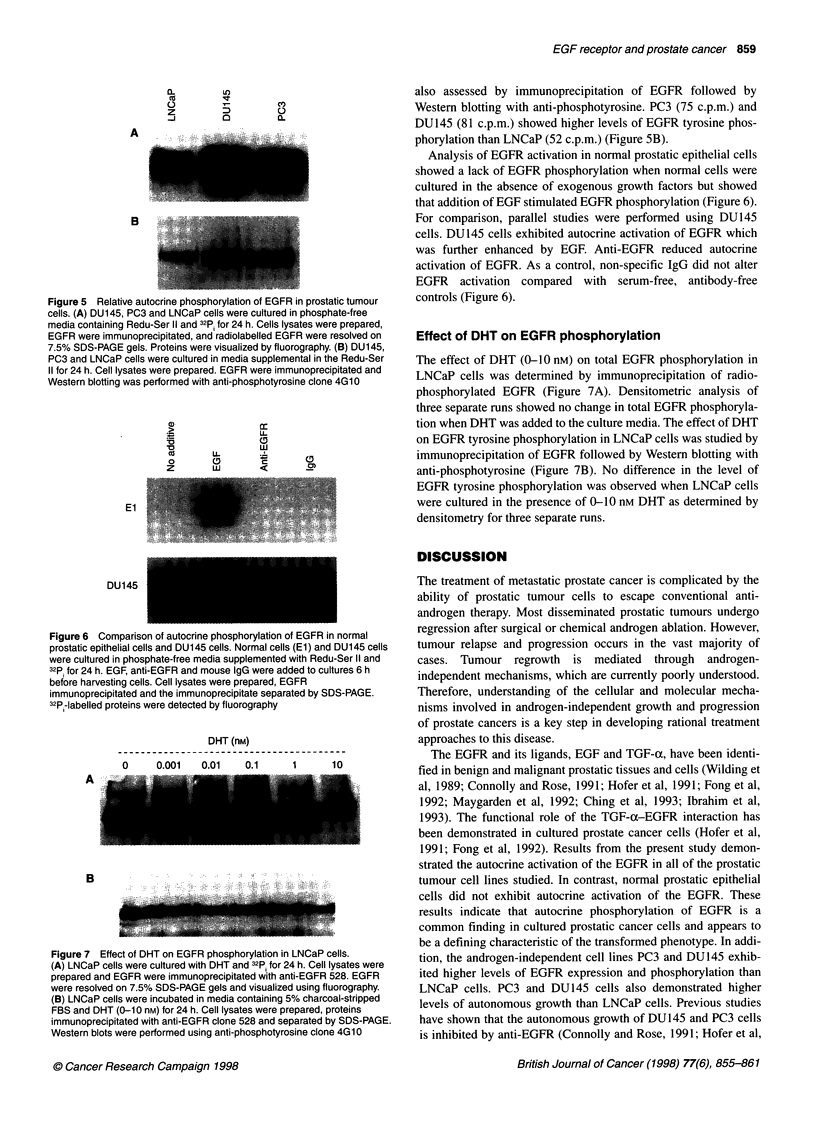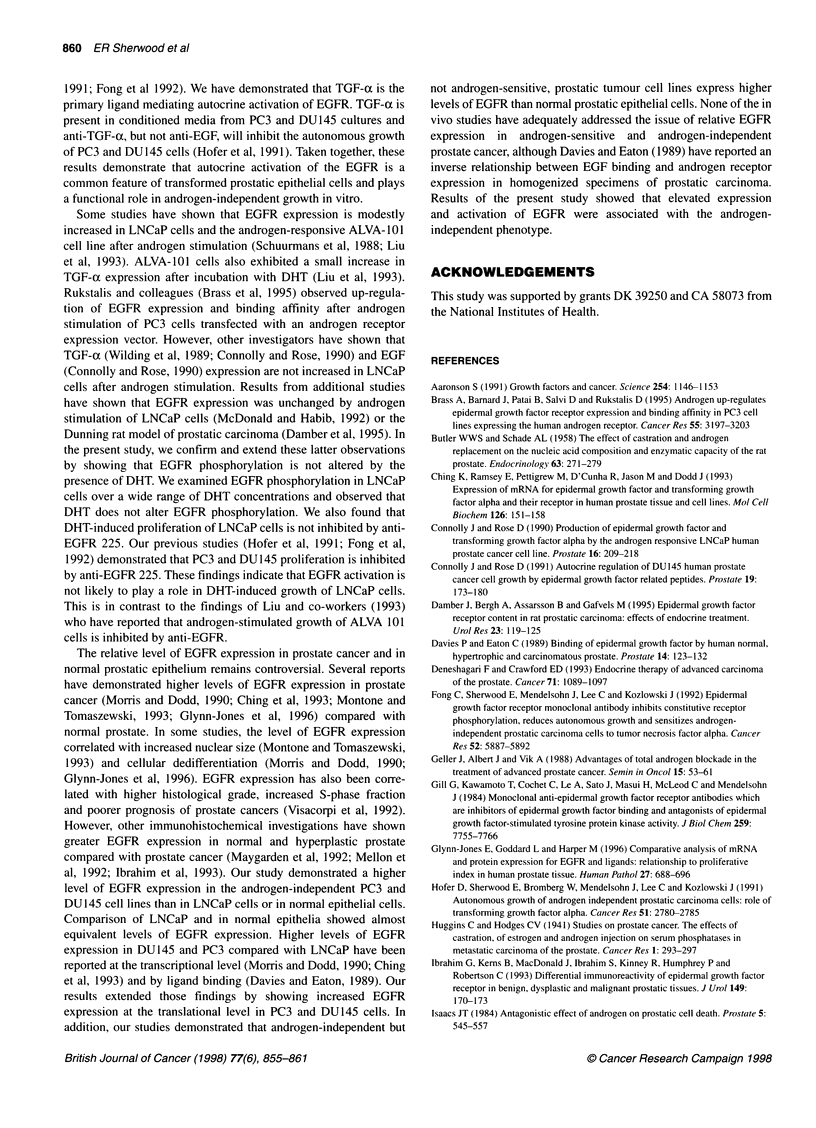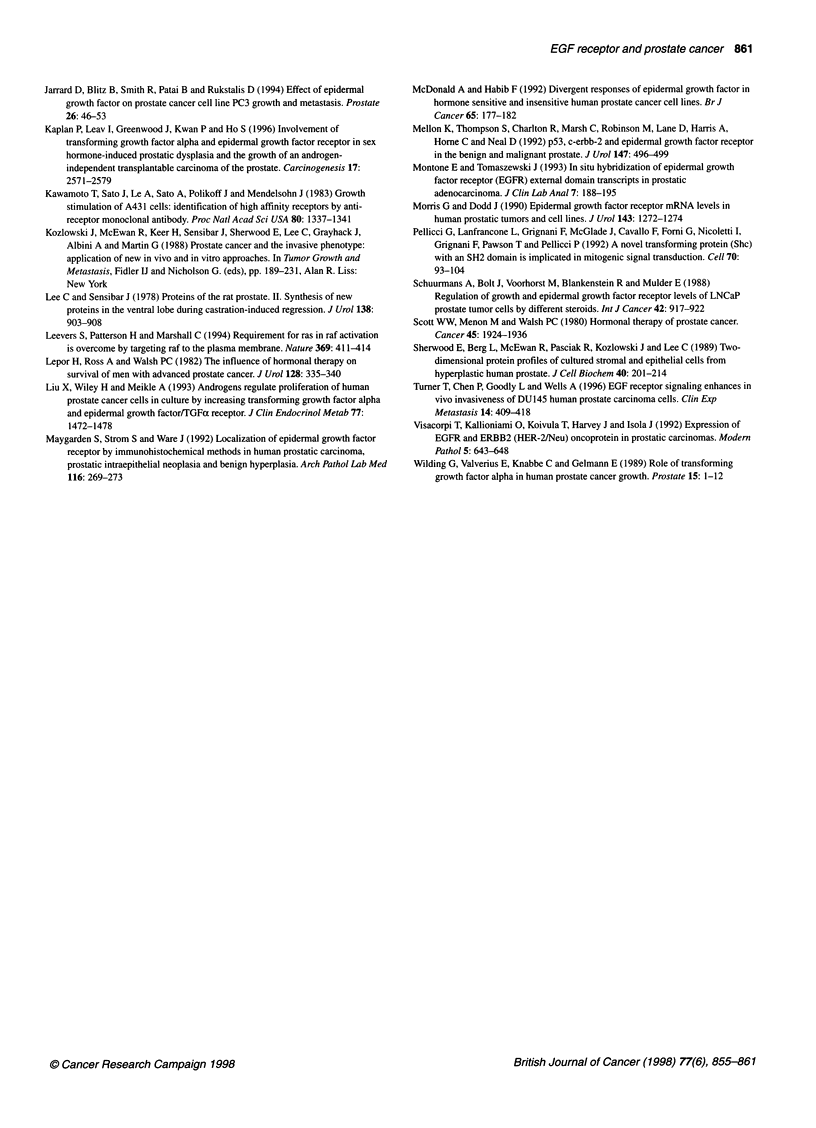# Epidermal growth factor receptor activation in androgen-independent but not androgen-stimulated growth of human prostatic carcinoma cells.

**DOI:** 10.1038/bjc.1998.142

**Published:** 1998-03

**Authors:** E. R. Sherwood, J. L. Van Dongen, C. G. Wood, S. Liao, J. M. Kozlowski, C. Lee

**Affiliations:** Department of Urology, Northwestern University Medical School, Chicago, IL 60611, USA.

## Abstract

**Images:**


*British Journal of Cancer* (1998) **77**(6), 855–861
© 1998 Cancer Research Campaign

### Epidermal growth factor receptor activation in androgen-independent but not androgen-stimulated growth of human prostatic carcinoma cells

**ER Sherwood[1], JL Van Dongen[1], CG Wood[1], S Liao[2], JM Kozlowski[1] and C Lee[1]**

[1]Urological Oncology Program, Department of Urology, Northwestern University Medical School, 303 East Chicago Avenue, Chicago, IL 60611, USA; [2]Ben May Institute and Department of Biochemistry and Molecular Biology, University of Chicago Medical Center, 5841 South Maryland Avenue, Chicago, IL 60637, USA

**Summary** These studies were undertaken to assess the relative expression and autocrine activation of the epidermal growth factor receptor (EGFR) in normal and transformed prostatic epithelial cells and to determine whether EGFR activation plays a functional role in androgen-stimulated growth of prostate cancer cells in vitro. EGFR expression was determined by Western blot analysis and ELISA immunoassays. Immunoprecipitation of radiophosphorylated EGFR and evaluation of tyrosine phosphorylation was used to assess EGFR activation. The human androgen-independent prostate cancer cell lines PC3 and DU145 exhibited higher levels of EGFR expression and autocrine phosphorylation than normal human prostatic epithelial cells or the human androgen-responsive prostate cancer cell line LNCaP. PC3 and DU145 cells also showed higher levels of autonomous growth under serum-free defined conditions. Normal prostatic epithelial cells expressed EGFR but did not exhibit detectable levels of EGFR phosphorylation when cultured in the absence of exogenous EGF. Addition of EGF stimulated EGFR phosphorylation and induced proliferation of normal cells. LNCaP cells exhibited autocrine phosphorylation of EGFR but did not undergo significant proliferation when cultured in the absence of exogenous growth factors. A biphasic growth curve was observed when LNCaP cells were cultured with dihydrotestosterone (DHT). Maximum proliferation occurred at 1 nM DHT with regression of the growth response at DHT concentrations greater than 1 nM. However, neither EGFR expression nor phosphorylation was altered in LNCaP cells after androgen stimulation. In addition, DHT-stimulated growth of LNCaP cells was not inhibited by anti-EGFR. These studies show that autocrine activation of EGFR is a common feature of prostatic carcinoma cells in contrast to normal epithelial cells. However, EGFR activation does not appear to play a functional role in androgen-stimulated growth of LNCaP cells in vitro.

**Keywords:** prostate cancer; androgen; epidermal growth factor receptors

The prostate gland is dependent on the presence of circulating androgens to maintain its normal structure and function (Butler and Schade, 1958; Lee and Sensibar, 1978; Isaacs, 1984). Likewise, prostatic neoplasms are androgen-sensitive tumours that undergo regression after chemical or surgical androgen ablation (Scott et al, 1980; Geller et al, 1988; Deneshagari and Crawford, 1993). However, the vast majority of disseminated prostatic adenocarcinomas recur and progress in the androgen-depleted environment (Lepor et al, 1982). Although new and more sophisticated methods of inducing androgen ablation have been developed in recent years, the basic premise of anti-androgen therapy in the treatment of prostate cancer has remained largely unchanged since the pioneering work of Huggins and Hodges in the 1940s (Huggins and Hodges, 1941). After recurrence in the androgen-depleted host, most disseminated, androgen-independent prostatic tumours are resistant to conventional chemotherapy and radiation treatment. Therefore, understanding of the mechanisms of androgen-independent growth of prostate cancers with the hope of developing effective interventions against metastatic androgen-independent tumours is a major area of interest in the field of prostate cancer research.

*Received 30 April 1997*
*Revised 1 July 1997*
*Accepted 8 July 1997*

*Correspondence to:* C Lee

The epidermal growth factor receptor (EGFR) is a 170-kDa transmembrane glycoprotein that has been identified in normal, hyperplastic and malignant prostatic epithelium (Maygarden et al, 1992; Ching et al, 1993; Ibrahim et al, 1993). The binding of EGF, TGF-$\alpha$ or amphiregulin results in tyrosine phosphorylation of EGFR and activation of downstream signal transduction pathways (Aaronson, 1991; Pellicci et al, 1992; Leevers et al, 1994). Results of several recent investigations have demonstrated increased TGF-$\alpha$ expression and activation of EGFR in human and non-human prostatic carcinomas (Hofer et al, 1991; Fong et al, 1992; Kaplan et al, 1996). Autocrine activation of EGFR has been proposed as a mechanism to support neoplastic growth and tissue invasiveness of transformed prostatic epithelial cells (Hofer et al, 1991; Fong et al, 1992; Jarrard et al, 1994; Turner et al, 1996). However, the relative level of EGFR expression in normal prostatic epithelial cells as well as in androgen-responsive and androgen-independent prostatic tumour cells has not been clearly defined. In addition, the functional role of EGFR activation in androgen-stimulated growth of prostate cancers has not been determined. The present studies were undertaken to define the relative expression and activation of EGFR in normal and malignant prostatic epithelial cells. In addition, EGFR expression and autocrine phosphorylation was determined in androgen-responsive and androgen-independent prostate cancer cells. Further studies were undertaken to determine the functional role of EGFR activation in androgen-stimulated growth of LNCaP cells in vitro.

### MATERIALS AND METHODS

### Cell lines

The androgen-independent prostatic carcinoma cell lines PC3 and DU145 were generously provided by Drs E Kaighn (Experimental Carcinogenesis Laboratory, NIH) and D Mickey (Duke University Medical Center, Durham, NO, USA) respectively. The androgen-responsive prostatic carcinoma cell line LNCaP was purchased from the American Type Culture Collection (Rockville, MD, USA). Stock cultures of the respective cell lines were maintained in RPMI-1640 medium containing 10% fetal bovine serum (FBS) and penicillin (100 U $ml^{-1}$)/streptomycin (100 ug $ml^{-1}$) (P/S). Normal prostatic epithelial cells were isolated from fresh prostatic tissue obtained from organ donors of less than 35 years of age and established in primary culture. Serial sections of prostatic tissue were examined microscopically after staining with haematoxylin and eosin to assure the absence of carcinoma or benign hyperplasia. Epithelial cells were isolated by enzymatic and mechanical dissociation of prostatic tissue followed by Percoll gradient centrifugation of the resulting cell suspension as previously described (Kozlowski et al, 1988; Sherwood et al, 1989). Isolated epithelial cells were cultivated in complete WAJC 404 medium which consisted of WAJC 404 medium supplemented with bovine pituitary extract (30 ug $ml^{-1}$, Upstate Biotechnology, Lake Placid, NY, USA), EGF (10 ng $ml^{-1}$, Upstate Biotechnology), cholera toxin (10 ng $ml^{-1}$, Sigma Chemical, St Louis, MO, USA), prolactin (3 ng $ml^{-1}$, Sigma), Redu-Ser II (insulin 5 μg $ml^{-1}$, transferrin 5 μg $ml^{-1}$, sodium selenite 5 ng $ml^{-1}$, bovine serum albumin 500 μg $ml^{-1}$, oleic acid 4.3 μg $ml^{-1}$, Upstate Biotechnology) and P/S. Normal epithelial cells were used in experiments after one or two serial passages. Primary cultures of prostatic epithelial cells are designated as E1 throughout the text.

### Antibodies and growth factors

Monoclonal antibodies 225 and 528 raised against the human epidermal growth factor receptor (EGFR) were generously provided by Dr J Mendelsohn (Memorial Sloan-Kettering Cancer Center) and were prepared as previously outlined (Kawamoto et al, 1983; Gill et al, 1984). Anti-EGFR clone LA22, monoclonal anti-phosphotyrosine clone 4G10 and EGF were purchased from Upstate Biotechnology. Dihydrotestosterone (DHT) was purchased from Sigma.

### Assessment of cell numbers

Cells (1–2 × $10^4$ per well) were plated in 24-well plates and allowed to adhere overnight (16–18 h). The following day, cultures were washed three times with phosphate-buffered saline (PBS, pH 7.4) and experimental samples were added to respective wells. Day 0 cell counts were performed to determine plating efficiency. Cells were cultured (37°C, 5% carbon dioxide) for 6 days and media were replaced on days 2 and 4. The cells were harvested using 0.25% trypsin/0.1% EDTA (Life Technologies, Grand Island, NY, USA) and counted using a Coulter counter (Coulter Electronics, Hialeah, FL, USA).

### Western blotting and ELISA immunoassays

For Western blotting, cells were plated (2 × $10^5$ per well) in six-well plates and grown to 80–90% confluency. The cells were harvested using 1.5 mM EDTA in PBS followed by gentle scraping with the plunger of a 1-ml syringe and were counted using a haemocytometer. The cells were washed (3×) with cold PBS, disrupted using lysis buffer (1% Triton X-100, 10 mM Tris-HCl (pH 7.0), 1.5 mM EDTA, 1 mM phenylmethylsulphonyl fluoride (PMSF), 1 mM sodium vanadate, 1 μg $ml^{-1}$ leupeptin, pepstatin and aprotinin) at a concentration of 1 × $10^7$ cells per 0.5 ml of lysis buffer, and the Triton-soluble supernatant was harvested after centrifugation at 14 000 *g* for 10 min. Protein concentration was determined using the Coomassie protein assay (Pierce Chemical, Rockford, IL, USA). Proteins (200 μg per lane) were separated by SDS-PAGE and electrophoretically transferred to nitrocellulose membranes (0.2 μm, Bio-Rad, Hercules, CA, USA) using a transfer buffer consisting of 192 mM glycine, 25 mM Tris-HCl and 20% methanol. Blots were blocked for 2–4 h in 5% non-fat dry

**Table 1** Proliferation of normal and transformed prostatic epithelial cells in the presence and absence of EGF

| Cell line | Cells per well × $10^{-4}$ | | | |
|---|---|---|---|---|
| | Day 0 | No EGF | EGF | Complete media |
| E1 | 1.2 ± 0.2 | 1.4 ± 0.3 | 3.6 ± 0.4* | 6.0 ± 0.4* |
| LNCaP | 1.1 ± 0.1 | 1.2 ± 0.4 | 2.5 ± 0.4* | 6.1 ± 0.5* |
| DU145 | 1.5 ± 0.3 | 4.2 ± 0.3* | 6.5 ± 0.7* | 13.9 ± 2.6* |
| PC3 | 1.0 ± 0.2 | 8.3 ± 1.2* | 9.9 ± 0.7* | 12.2 ± 1.8* |

Cells were cultured under defined conditions in 24-well plates for 6 days and counted with a Coulter counter. Day 0 cell counts were performed to determine plating efficiency and provide a reference point for assessing cellular proliferation. In additional wells, cells were cultured in defined EGF-free medium or with EGF (20 ng $ml^{-1}$) to assess EGF-induced proliferation. As a positive control, cells were cultured with RPMI-1640 media containing 10% FBS (PC3, DU145, LNCaP) or complete WAJC 404 (normal epithelial cells). E1 refers to primary cultures of normal prostatic epithelial cells. $n$ = 8–12 per group. *$P < 0.05$ compared with Day 0 control.

**Figure 1** Effect of DHT on the proliferation of LNCaP cells. LNCaP cells (2 × $10^4$ per well) were plated in 24-well plates and allowed to adhere overnight. Cells were cultured for 6 days in RPMI-1640 media supplemented with 10% charcoal-stripped FBS and varying concentrations of DHT as indicated. Anti-EGFR clone 528 (20 nM) was added to additional wells to determine the effect of anti-EGFR on DHT-stimulated growth of LNCaP cells. Non-specific IgG served as a control. Cells were detached from the culture surface with trypsin/EDTA and counted with a Coulter counter to determine cell numbers in each group. Values represent the mean ± s.e.m. $n$ = 8–12 per group

© Cancer Research Campaign 1998

**Figure 2** Relative expression of EGFR by normal (E1) and malignant prostatic epithelial cells. Cell lysates were prepared and 200 µg of protein from each cell type was separated by SDS-PAGE. EGFR were detected by Western blotting using monoclonal anti-EGFR clone LA22. Molecular weight markers are indicated in kDa

**Table 2** Relative expression of EGFR by prostatic cell lines

| Cell line | Densitometry (c.p.m.) | ELISA (receptors per cell × $10^{-4}$) |
|---|---|---|
| PC3 | 254 ± 28* | 20.9 ± 3.1* |
| DU145 | 342 ± 39* | 25.2 ± 3.9* |
| LNCaP | 68 ± 6 | 6.3 ± 0.9 |
| E1 | 89 ± 7 | 7.9 ± 0.5 |

EGFR levels were measured by Western blotting. Proteins (200 µg per lane) were separated by SDS-PAGE, immunoblotted with anti-EGFR 528 and visualized using the ECL system. Band intensity was quantitated by densitometry and results reflect the mean ± s.e.m. for three separate determinations. For the ELISA, cells were cultured in 10% FBS, cell lysates were prepared and the assay was performed as outlined in Materials and methods. The results reflect the mean ± s.e.m. for at least six separate wells for each cell type. *Significantly ($P < 0.05$) greater EGFR than LNCaP cells and normal prostatic epithelial cells.

milk reconstituted in PBS and incubated at 25°C with primary antibody for 16–18 h. The blots were washed (3×) with TTBS (0.05% Tween 20, 50 mM Tris-HC1, 200 mM sodium cholride) and incubated at 25°C with horseradish peroxidase (HRP)-conjugated secondary antibody for 1 h. Immunoreactive proteins were visualized using the enhanced chemiluminescence (ECL) system (Amersham, Arlington Heights, IL, USA). Band intensities were quantitated using the Ambis Image Acquisition and Analysis System (Ambis, San Diego, CA, USA).

For the ELISA assay, samples were prepared as outlined above for Western blotting except that manufacturer-supplied antigen extraction buffer was used to prepare cell lysates. Cells (2 × $10^7$ per 0.5 ml of antigen extraction buffer) were lysed, centrifuged (14 000 *g* for 10 min) and the supernatant was harvested. Samples were diluted with antigen extraction buffer to achieve protein concentrations of 0.4–50 µg $ml^{-1}$. The human EGFR ELISA was purchased from Oncogene Science (Uniondale, NY, USA) and the assay was performed according to the manufacturer's instructions. Briefly, samples (0.4–50 µg of protein $ml^{-1}$) were added to 96-well microtitre wells precoated with monoclonal anti-EGFR and incubated at 37°C for 3 h. EGFR standards of 0–80 fmol $ml^{-1}$ as provided by the manufacturer were added to wells in duplicate for development of a standard curve. The wells were washed six times using a plate washer (Bio-Rad Model 1250) followed by addition of rabbit anti-EGFR and incubation at 25°C for 1 h. After washing, HRP-conjugated goat anti-rabbit IgG was added and plates were incubated at 25°C for 1 h. A sulphuric acid solution (2.5 N) was added to each well to stop the peroxidase reaction, and samples were analysed with a microplate reader (Bio-Rad Model 3550) at 490 nM.

### Labelling of proteins with $^{32}P_i$

Cells were plated (3 × $10^5$ cells per well) in six-well plates and allowed to adhere overnight. The following day, cells were washed three times with PBS and cultured in serum-free, growth factor-free media for 24 h. Cells were then washed three times with phosphate-free Dulbecco's modified Eagle Medium (DMEM) and cultured in phosphate-free DMEM containing $^{32}P_i$ (0.4 µCi per well, Amersham) and experimental additives for 24 h. Cells were then washed three times with PBS and 2 ml of lysis buffer was added to each well. Samples were harvested and cleared by centrifugation (2000 *g* for 10 min). In additional wells, cells were detached from the culture surface using 0.25% trypsin/0.1% EDTA and counted with a Coulter counter to quantitate cell numbers in each group. Immunoprecipitation of radiophosphorylated EGFR was performed using 500 µg of protein from each group.

### Immunoprecipitation

Protein A Sepharose 4B Fast Flow beads (Sigma Chemical) were combined with anti-EGFR 528 by incubating 100 µl of 20% (v/v) beads with 2 µg of antibody at 4°C for 16–18 h. Beads were washed three times with cold PBS and added to cell lysates (1 × $10^6$ cells per sample). Samples were incubated at 4°C for 16–18 h with shaking, and beads were washed three times with cold Triton-free lysis buffer followed by elution of proteins with Laemmli buffer (0.5 M Tris-HCl, 2% sodium dodecyl sulphate) and boiling. Immunoprecipitated proteins were separated by SDS-PAGE.

### Statistics

Statistical analyses were performed using one-way analysis of variance followed by Student's t-test. A value of $P < 0.05$ was considered to be statistically significant.

### RESULTS

### Autonomous and EGF-stimulated growth of benign and malignant prostatic epithelial cells

The proliferation of benign and malignant prostatic epithelial cells was evaluated in the presence and absence of EGF (Table 1). Normal prostatic epithelial cells established in primary culture (E1) did not exhibit significant proliferation when cultured in serum-free WAJC 404 medium devoid of EGF. Cultivation of normal cells in defined media supplemented with EGF or in complete WAJC 404 medium resulted in twofold and fivefold ($P < 0.05$) increases in cell number, respectively, compared with EGF-free controls. LNCaP cells did not exhibit growth when cultured in EGF-free medium. However, addition of EGF more than doubled ($P < 0.05$) LNCaP proliferation compared with EGF-free controls and a greater than fourfold increase in LNCaP proliferation was observed when cells were cultured with medium containing 10% fetal bovine serum (FBS). DU145 and PC3 cells exhibited significant

© Cancer Research Campaign 1998

**Figure 3** Effect of DHT and EGF on EGFR expression in prostatic carcinoma cells. Cells were cultured for 24 h in media containing Redu-Ser II alone or Redu-Ser II supplemented with EGF (30 ng $ml^{-1}$) or DHT (1 nM). Cells were harvested, quantitated and lysed ($1 \times 10^7$ per 0.5 ml of lysis buffer). Proteins (200 μg per lane) were separated by SDS-PAGE and detected by Western blotting with anti-EGFR clone LA22

**Figure 4** Lack of DHT-induced alteration in EGFR expression in LNCaP cells. LNCaP cells were cultured in media supplemented with 10% charcoal-stripped FBS and DHT (0–10 nM) for 24 h. Cell lysates were prepared and proteins (200 μg per lane) were separated by SDS-PAGE. EGFR were detected by Western blotting. Equivalent protein (200 μg per well) from PC3 and DU145 cells were run in parallel for comparison

3.4-fold and 7.6-fold increases in cell number, respectively, compared with day 0 controls when cultured in defined, growth factor-free media. Addition of EGF further enhanced the growth of DU145 and PC3. The highest level of growth was observed when PC3 and DU145 were cultured in media containing 10% FBS.

Additional studies were undertaken to assess the growth response of LNCaP cells to DHT and to determine the effect of anti-EGFR on DHT-stimulated proliferation of LNCaP cells (Figure 1). A biphasic growth curve was observed with peak cell growth occurring at a DHT concentration of 1 nM with regression of DHT-enhanced proliferation at DHT concentrations greater than 1 nM. Addition of anti-EGFR 225 to LNCaP cells cultured with DHT did not significantly alter LNCaP cell proliferation compared with cells cultured in the absence of anti-EGFR. As a control, LNCaP cells were also cultured with non-specific IgG, which also did not alter DHT-enhanced proliferation of LNCaP cells.

### EGF receptor expression in normal and transformed prostatic epithelial cells

The relative expression of EGFR in different prostatic cells was determined by Western blot analysis (Figure 2). The androgen-independent cell lines PC3 and DU145 showed a higher level of EGFR expression than normal epithelial cells (E1) or LNCaP

**Table 3** Relative expression of EGFR in LNCaP cells incubated with DHT

| DHT (nM) | Densitometry (c.p.m.) | ELISA (receptors per cell × $10^{-4}$) |
|---|---|---|
| 0 | 92 ± 5 | 4.6 ± 0.6 |
| 0.001 | 80 ± 6 | 4.2 ± 0.8 |
| 0.01 | 86 ± 9 | 5.6 ± 0.5 |
| 0.1 | 90 ± 11 | 4.3 ± 1.1 |
| 1 | 96 ± 7 | 4.0 ± 0.8 |
| 10 | 98 ± 12 | 4.8 ± 0.7 |

LNCaP cells were cultured in the presence of DHT at concentrations of 0–10 nM for 24 h. Cells were harvested, counted and lysates were prepared. Proteins (200 μg per lane) were separated by SDS-PAGE, and EGFR bands were visualized by immunoblotting with anti-EGFR. EGFR expression was quantitated by densitometric analysis of Western blots. EGFR in cell lysates was also quantitated by ELISA. ELISA and Western blot results represent data from at least three separate determinations for each group. Values are expressed as the mean ± s.e.m.

cells. Band intensity of Western blots were quantitated by densitometry (Table 2). Densitometric analysis showed that PC3 and DU145 expressed 3.7-fold and 5.0-fold higher levels of EGFR than LNCaP respectively. Compared with normal epithelial cells, PC3 and DU145 expressed 2.9-fold and 3.8-fold greater levels of EGFR. Comparison of EGFR expression between LNCaP and normal epithelial cells did not show a statistically significant difference.

The relative level of EGFR expression in prostatic cells was also quantitated by ELISA (Table 2). The level of EGFR expression in PC3 and DU145, cells was 3.3-fold and 4.2-fold greater, respectively, than that observed in LNCaP. Compared with normal epithelial cells, 2.6-fold and 3.3-fold greater levels of EGFR expression were observed in PC3 and DU145 cells, respectively.

### Effect of DHT and EGF on EGFR expression in prostatic tumour cells

Examination of EGFR expression in the presence of exogenous EGF or DHT showed that EGF down-regulated EGFR expression in LNCaP and PC3 cells while DHT at 1 nM had no effect (Figure 3). Dose–response studies showed that DHT at concentrations of 0.001–10 nM did not alter EGFR expression in androgen-responsive LNCaP cells (Figure 4). For comparison, PC3 and DU145 cells were run in parallel lanes. As previously shown, PC3 and DU145 cells expressed higher levels of EGFR than LNCaP. The quantitative analysis of EGFR bands on Western blots by densitometry or assessment of EGFR content by ELISA also showed that DHT at 0.001–10 nM did not significantly alter EGFR expression in androgen-responsive LNCaP cells (Table 3).

### Phosphorylation of EGFR in benign and malignant prostatic epithelial cells

Immunoprecipitation studies were conducted after labelling of EGFR with $^{32}P_i$ under serum-free, growth factor-free conditions (Figure 5A). The prostatic tumour cell lines PC3, DU145 and LNCaP exhibited autocrine phosphorylation of the EGFR (Figure 5A). Densitometric analysis showed that DU145 (212 c.p.m.) and PC3 (182 c.p.m.) possessed higher levels of autocrine EGFR phosphorylation than LNCaP (72 c.p.m.). The relative tyrosine phosphorylation of EGFR in LNCaP, PC3 and DU145 cells was

© Cancer Research Campaign 1998

**Figure 5** Relative autocrine phosphorylation of EGFR in prostatic tumour cells. (**A**) DU145, PC3 and LNCaP cells were cultured in phosphate-free media containing Redu-Ser II and $^{32}P_i$ for 24 h. Cells lysates were prepared, EGFR were immunoprecipitated, and radiolabelled EGFR were resolved on 7.5% SDS-PAGE gels. Proteins were visualized by fluorography. (**B**) DU145, PC3 and LNCaP cells were cultured in media supplemental in the Redu-Ser II for 24 h. Cell lysates were prepared. EGFR were immunoprecipitated and Western blotting was performed with anti-phosphotyrosine clone 4G10

**Figure 6** Comparison of autocrine phosphorylation of EGFR in normal prostatic epithelial cells and DU145 cells. Normal cells (E1) and DU145 cells were cultured in phosphate-free media supplemented with Redu-Ser II and $^{32}P_i$ for 24 h. EGF, anti-EGFR and mouse IgG were added to cultures 6 h before harvesting cells. Cell lysates were prepared, EGFR immunoprecipitated and the immunoprecipitate separated by SDS-PAGE. $^{32}P_i$-labelled proteins were detected by fluorography

**Figure 7** Effect of DHT on EGFR phosphorylation in LNCaP cells. (**A**) LNCaP cells were cultured with DHT and $^{32}P_i$ for 24 h. Cell lysates were prepared and EGFR were immunoprecipitated with anti-EGFR 528. EGFR were resolved on 7.5% SDS-PAGE gels and visualized using fluorography. (**B**) LNCaP cells were incubated in media containing 5% charcoal-stripped FBS and DHT (0–10 nM) for 24 h. Cell lysates were prepared, proteins immunoprecipitated with anti-EGFR clone 528 and separated by SDS-PAGE. Western blots were performed using anti-phosphotyrosine clone 4G10

also assessed by immunoprecipitation of EGFR followed by Western blotting with anti-phosphotyrosine. PC3 (75 c.p.m.) and DU145 (81 c.p.m.) showed higher levels of EGFR tyrosine phosphorylation than LNCaP (52 c.p.m.) (Figure 5B).

Analysis of EGFR activation in normal prostatic epithelial cells showed a lack of EGFR phosphorylation when normal cells were cultured in the absence of exogenous growth factors but showed that addition of EGF stimulated EGFR phosphorylation (Figure 6). For comparison, parallel studies were performed using DU145 cells. DU145 cells exhibited autocrine activation of EGFR which was further enhanced by EGF. Anti-EGFR reduced autocrine activation of EGFR. As a control, non-specific IgG did not alter EGFR activation compared with serum-free, antibody-free controls (Figure 6).

### Effect of DHT on EGFR phosphorylation

The effect of DHT (0–10 nM) on total EGFR phosphorylation in LNCaP cells was determined by immunoprecipitation of radiophosphorylated EGFR (Figure 7A). Densitometric analysis of three separate runs showed no change in total EGFR phosphorylation when DHT was added to the culture media. The effect of DHT on EGFR tyrosine phosphorylation in LNCaP cells was studied by immunoprecipitation of EGFR followed by Western blotting with anti-phosphotyrosine (Figure 7B). No difference in the level of EGFR tyrosine phosphorylation was observed when LNCaP cells were cultured in the presence of 0–10 nM DHT as determined by densitometry for three separate runs.

### DISCUSSION

The treatment of metastatic prostate cancer is complicated by the ability of prostatic tumour cells to escape conventional anti-androgen therapy. Most disseminated prostatic tumours undergo regression after surgical or chemical androgen ablation. However, tumour relapse and progression occurs in the vast majority of cases. Tumour regrowth is mediated through androgen-independent mechanisms, which are currently poorly understood. Therefore, understanding of the cellular and molecular mechanisms involved in androgen-independent growth and progression of prostate cancers is a key step in developing rational treatment approaches to this disease.

The EGFR and its ligands, EGF and TGF-α, have been identified in benign and malignant prostatic tissues and cells (Wilding et al, 1989; Connolly and Rose, 1991; Hofer et al, 1991; Fong et al, 1992; Maygarden et al, 1992; Ching et al, 1993; Ibrahim et al, 1993). The functional role of the TGF-α–EGFR interaction has been demonstrated in cultured prostate cancer cells (Hofer et al, 1991; Fong et al, 1992). Results from the present study demonstrated the autocrine activation of the EGFR in all of the prostatic tumour cell lines studied. In contrast, normal prostatic epithelial cells did not exhibit autocrine activation of the EGFR. These results indicate that autocrine phosphorylation of EGFR is a common finding in cultured prostatic cancer cells and appears to be a defining characteristic of the transformed phenotype. In addition, the androgen-independent cell lines PC3 and DU145 exhibited higher levels of EGFR expression and phosphorylation than LNCaP cells. PC3 and DU145 cells also demonstrated higher levels of autonomous growth than LNCaP cells. Previous studies have shown that the autonomous growth of DU145 and PC3 cells is inhibited by anti-EGFR (Connolly and Rose, 1991; Hofer et al,

© Cancer Research Campaign 1998

1991; Fong et al 1992). We have demonstrated that TGF-α is the primary ligand mediating autocrine activation of EGFR. TGF-α is present in conditioned media from PC3 and DU145 cultures and anti-TGF-α, but not anti-EGF, will inhibit the autonomous growth of PC3 and DU145 cells (Hofer et al, 1991). Taken together, these results demonstrate that autocrine activation of the EGFR is a common feature of transformed prostatic epithelial cells and plays a functional role in androgen-independent growth in vitro.

Some studies have shown that EGFR expression is modestly increased in LNCaP cells and the androgen-responsive ALVA-101 cell line after androgen stimulation (Schuurmans et al, 1988; Liu et al, 1993). ALVA-101 cells also exhibited a small increase in TGF-α expression after incubation with DHT (Liu et al, 1993). Rukstalis and colleagues (Brass et al, 1995) observed up-regulation of EGFR expression and binding affinity after androgen stimulation of PC3 cells transfected with an androgen receptor expression vector. However, other investigators have shown that TGF-α (Wilding et al, 1989; Connolly and Rose, 1990) and EGF (Connolly and Rose, 1990) expression are not increased in LNCaP cells after androgen stimulation. Results from additional studies have shown that EGFR expression was unchanged by androgen stimulation of LNCaP cells (McDonald and Habib, 1992) or the Dunning rat model of prostatic carcinoma (Damber et al, 1995). In the present study, we confirm and extend these latter observations by showing that EGFR phosphorylation is not altered by the presence of DHT. We examined EGFR phosphorylation in LNCaP cells over a wide range of DHT concentrations and observed that DHT does not alter EGFR phosphorylation. We also found that DHT-induced proliferation of LNCaP cells is not inhibited by anti-EGFR 225. Our previous studies (Hofer et al, 1991; Fong et al, 1992) demonstrated that PC3 and DU145 proliferation is inhibited by anti-EGFR 225. These findings indicate that EGFR activation is not likely to play a role in DHT-induced growth of LNCaP cells. This is in contrast to the findings of Liu and co-workers (1993) who have reported that androgen-stimulated growth of ALVA 101 cells is inhibited by anti-EGFR.

The relative level of EGFR expression in prostate cancer and in normal prostatic epithelium remains controversial. Several reports have demonstrated higher levels of EGFR expression in prostate cancer (Morris and Dodd, 1990; Ching et al, 1993; Montone and Tomaszewski, 1993; Glynn-Jones et al, 1996) compared with normal prostate. In some studies, the level of EGFR expression correlated with increased nuclear size (Montone and Tomaszewski, 1993) and cellular dedifferentiation (Morris and Dodd, 1990; Glynn-Jones et al, 1996). EGFR expression has also been correlated with higher histological grade, increased S-phase fraction and poorer prognosis of prostate cancers (Visacorpi et al, 1992). However, other immunohistochemical investigations have shown greater EGFR expression in normal and hyperplastic prostate compared with prostate cancer (Maygarden et al, 1992; Mellon et al, 1992; Ibrahim et al, 1993). Our study demonstrated a higher level of EGFR expression in the androgen-independent PC3 and DU145 cell lines than in LNCaP cells or in normal epithelial cells. Comparison of LNCaP and in normal epithelia showed almost equivalent levels of EGFR expression. Higher levels of EGFR expression in DU145 and PC3 compared with LNCaP have been reported at the transcriptional level (Morris and Dodd, 1990; Ching et al, 1993) and by ligand binding (Davies and Eaton, 1989). Our results extended those findings by showing increased EGFR expression at the translational level in PC3 and DU145 cells. In addition, our studies demonstrated that androgen-independent but not androgen-sensitive, prostatic tumour cell lines express higher levels of EGFR than normal prostatic epithelial cells. None of the in vivo studies have adequately addressed the issue of relative EGFR expression in androgen-sensitive and androgen-independent prostate cancer, although Davies and Eaton (1989) have reported an inverse relationship between EGF binding and androgen receptor expression in homogenized specimens of prostatic carcinoma. Results of the present study showed that elevated expression and activation of EGFR were associated with the androgen-independent phenotype.

### ACKNOWLEDGEMENTS

This study was supported by grants DK 39250 and CA 58073 from the National Institutes of Health.

### REFERENCES

Aaronson S (1991) Growth factors and cancer. *Science* **254**: 1146–1153

Brass A, Barnard J, Patai B, Salvi D and Rukstalis D (1995) Androgen up-regulates epidermal growth factor receptor expression and binding affinity in PC3 cell lines expressing the human androgen receptor. *Cancer Res* **55**: 3197–3203

Butler WWS and Schade AL (1958) The effect of castration and androgen replacement on the nucleic acid composition and enzymatic capacity of the rat prostate. *Endocrinology* **63**: 271–279

Ching K, Ramsey E, Pettigrew M, D'Cunha R, Jason M and Dodd J (1993) Expression of mRNA for epidermal growth factor and transforming growth factor alpha and their receptor in human prostate tissue and cell lines. *Mol Cell Biochem* **126**: 151–158

Connolly J and Rose D (1990) Production of epidermal growth factor and transforming growth factor alpha by the androgen responsive LNCaP human prostate cancer cell line. *Prostate* **16**: 209–218

Connolly J and Rose D (1991) Autocrine regulation of DU145 human prostate cancer cell growth by epidermal growth factor related peptides. *Prostate* **19**: 173–180

Damber J, Bergh A, Assarsson B and Gafvels M (1995) Epidermal growth factor receptor content in rat prostatic carcinoma: effects of endocrine treatment. *Urol Res* **23**: 119–125

Davies P and Eaton C (1989) Binding of epidermal growth factor by human normal, hypertrophic and carcinomatous prostate. *Prostate* **14**: 123–132

Deneshagari F and Crawford ED (1993) Endocrine therapy of advanced carcinoma of the prostate. *Cancer* **71**: 1089–1097

Fong C, Sherwood E, Mendelsohn J, Lee C and Kozlowski J (1992) Epidermal growth factor receptor monoclonal antibody inhibits constitutive receptor phosphorylation, reduces autonomous growth and sensitizes androgen-independent prostatic carcinoma cells to tumor necrosis factor alpha. *Cancer Res* **52**: 5887–5892

Geller J, Albert J and Vik A (1988) Advantages of total androgen blockade in the treatment of advanced prostate cancer. *Semin in Oncol* **15**: 53–61

Gill G, Kawamoto T, Cochet C, Le A, Sato J, Masui H, McLeod C and Mendelsohn J (1984) Monoclonal anti-epidermal growth factor receptor antibodies which are inhibitors of epidermal growth factor binding and antagonists of epidermal growth factor-stimulated tyrosine protein kinase activity. *J Biol Chem* **259**: 7755–7766

Glynn-Jones E, Goddard L and Harper M (1996) Comparative analysis of mRNA and protein expression for EGFR and ligands: relationship to proliferative index in human prostate tissue. *Human Pathol* **27**: 688–696

Hofer D, Sherwood E, Bromberg W, Mendelsohn J, Lee C and Kozlowski J (1991) Autonomous growth of androgen independent prostatic carcinoma cells: role of transforming growth factor alpha. *Cancer Res* **51**: 2780–2785

Huggins C and Hodges CV (1941) Studies on prostate cancer. The effects of castration, of estrogen and androgen injection on serum phosphatases in metastatic carcinoma of the prostate. *Cancer Res* **1**: 293–297

Ibrahim G, Kerns B, MacDonald J, Ibrahim S, Kinney R, Humphrey P and Robertson C (1993) Differential immunoreactivity of epidermal growth factor receptor in benign, dysplastic and malignant prostatic tissues. *J Urol* **149**: 170–173

Isaacs JT (1984) Antagonistic effect of androgen on prostatic cell death. *Prostate* **5**: 545–557

© Cancer Research Campaign 1998

Jarrard D, Blitz B, Smith R, Patai B and Rukstalis D (1994) Effect of epidermal growth factor on prostate cancer cell line PC3 growth and metastasis. *Prostate* **26**: 46–53

Kaplan P, Leav I, Greenwood J, Kwan P and Ho S (1996) Involvement of transforming growth factor alpha and epidermal growth factor receptor in sex hormone-induced prostatic dysplasia and the growth of an androgen-independent transplantable carcinoma of the prostate. *Carcinogenesis* **17**: 2571–2579

Kawamoto T, Sato J, Le A, Sato A, Polikoff J and Mendelsohn J (1983) Growth stimulation of A431 cells: identification of high affinity receptors by anti-receptor monoclonal antibody. *Proc Natl Acad Sci USA* **80**: 1337–1341

Kozlowski J, McEwan R, Keer H, Sensibar J, Sherwood E, Lee C, Grayhack J, Albini A and Martin G (1988) Prostate cancer and the invasive phenotype: application of new in vivo and in vitro approaches. In *Tumor Growth and Metastasis*, Fidler IJ and Nicholson G. (eds), pp. 189–231, Alan R. Liss: New York

Lee C and Sensibar J (1978) Proteins of the rat prostate. II. Synthesis of new proteins in the ventral lobe during castration-induced regression. *J Urol* **138**: 903–908

Leevers S, Patterson H and Marshall C (1994) Requirement for ras in raf activation is overcome by targeting raf to the plasma membrane. *Nature* **369**: 411–414

Lepor H, Ross A and Walsh PC (1982) The influence of hormonal therapy on survival of men with advanced prostate cancer. *J Urol* **128**: 335–340

Liu X, Wiley H and Meikle A (1993) Androgens regulate proliferation of human prostate cancer cells in culture by increasing transforming growth factor alpha and epidermal growth factor/TGFα receptor. *J Clin Endocrinol Metab* **77**: 1472–1478

Maygarden S, Strom S and Ware J (1992) Localization of epidermal growth factor receptor by immunohistochemical methods in human prostatic carcinoma, prostatic intraepithelial neoplasia and benign hyperplasia. *Arch Pathol Lab Med* **116**: 269–273

McDonald A and Habib F (1992) Divergent responses of epidermal growth factor in hormone sensitive and insensitive human prostate cancer cell lines. *Br J Cancer* **65**: 177–182

Mellon K, Thompson S, Charlton R, Marsh C, Robinson M, Lane D, Harris A, Horne C and Neal D (1992) p53, c-erbb-2 and epidermal growth factor receptor in the benign and malignant prostate. *J Urol* **147**: 496–499

Montone E and Tomaszewski J (1993) In situ hybridization of epidermal growth factor receptor (EGFR) external domain transcripts in prostatic adenocarcinoma. *J Clin Lab Anal* **7**: 188–195

Morris G and Dodd J (1990) Epidermal growth factor receptor mRNA levels in human prostatic tumors and cell lines. *J Urol* **143**: 1272–1274

Pellicci G, Lanfrancone L, Grignani F, McGlade J, Cavallo F, Forni G, Nicoletti I, Grignani F, Pawson T and Pellicci P (1992) A novel transforming protein (Shc) with an SH2 domain is implicated in mitogenic signal transduction. *Cell* **70**: 93–104

Schuurmans A, Bolt J, Voorhorst M, Blankenstein R and Mulder E (1988) Regulation of growth and epidermal growth factor receptor levels of LNCaP prostate tumor cells by different steroids. *Int J Cancer* **42**: 917–922

Scott WW, Menon M and Walsh PC (1980) Hormonal therapy of prostate cancer. *Cancer* **45**: 1924–1936

Sherwood E, Berg L, McEwan R, Pasciak R, Kozlowski J and Lee C (1989) Two-dimensional protein profiles of cultured stromal and epithelial cells from hyperplastic human prostate. *J Cell Biochem* **40**: 201–214

Turner T, Chen P, Goodly L and Wells A (1996) EGF receptor signaling enhances in vivo invasiveness of DU145 human prostate carcinoma cells. *Clin Exp Metastasis* **14**: 409–418

Visacorpi T, Kallioniami O, Koivula T, Harvey J and Isola J (1992) Expression of EGFR and ERBB2 (HER-2/Neu) oncoprotein in prostatic carcinomas. *Modern Pathol* **5**: 643–648

Wilding G, Valverius E, Knabbe C and Gelmann E (1989) Role of transforming growth factor alpha in human prostate cancer growth. *Prostate* **15**: 1–12

© Cancer Research Campaign 1998